# Air Pollution from Industrial Swine Operations and Blood Pressure of Neighboring Residents

**DOI:** 10.1289/ehp.1205109

**Published:** 2012-10-28

**Authors:** Steve Wing, Rachel Avery Horton, Kathryn M. Rose

**Affiliations:** 1 Department of Epidemiology, University of North Carolina–Chapel Hill, Chapel Hill, North Carolina, USA; 2 Health Sciences Research, SRA International Inc., Durham, North Carolina, USA

**Keywords:** agriculture, air pollution, community-based participatory research, environmental justice, epidemiology, health disparities, odors, psychosocial stress

## Abstract

Background: Industrial swine operations emit odorant chemicals including ammonia, hydrogen sulfide (H_2_S), and volatile organic compounds. Malodor and pollutant concentrations have been associated with self-reported stress and altered mood in prior studies.

Objectives: We conducted a repeated-measures study of air pollution, stress, and blood pressure in neighbors of swine operations.

Methods: For approximately 2 weeks, 101 nonsmoking adult volunteers living near industrial swine operations in 16 neighborhoods in eastern North Carolina sat outdoors for 10 min twice daily at preselected times. Afterward, they reported levels of hog odor on a 9-point scale and measured their blood pressure twice using an automated oscillometric device. During the same 2- to 3-week period, we measured ambient levels of H_2_S and PM_10_ at a central location in each neighborhood. Associations between systolic and diastolic blood pressure (SBP and DBP, respectively) and pollutant measures were estimated using fixed-effects (conditional) linear regression with adjustment for time of day.

Results: PM_10_ showed little association with blood pressure. DBP [β (SE)] increased 0.23 (0.08) mmHg per unit of reported hog odor during the 10 min outdoors and 0.12 (0.08) mmHg per 1-ppb increase of H_2_S concentration in the same hour. SBP increased 0.10 (0.12) mmHg per odor unit and 0.29 (0.12) mmHg per 1-ppb increase of H_2_S in the same hour. Reported stress was strongly associated with BP; adjustment for stress reduced the odor–DBP association, but the H_2_S–SBP association changed little.

Conclusions: Like noise and other repetitive environmental stressors, malodors may be associated with acute blood pressure increases that could contribute to development of chronic hypertension.

The rapid global expansion of confined animal feeding operations (CAFOs) has created environmental health concerns at local, regional, and global scales, including infectious and respiratory diseases, reduced quality of life, impacts on the built environment, and environmental injustice (Pew Commission on Industrial Food Animal Production 2008). CAFO airborne emissions, including ammonia, hydrogen sulfide (H_2_S), volatile organic compounds, and endotoxins, originate from confinement buildings, waste storage areas, and land application of animal waste (National Research Council 2003).

North Carolina experienced a rapid transformation of swine production during the 1980s and 1990s. The number of producers declined, the size of operations grew, the swine population increased from approximately 2.5 million to 10 million, and production shifted to the eastern coastal plain region of the state (Furuseth 1997). In North Carolina, swine CAFOs are concentrated in low-income communities of color (mostly African American), where older housing and lack of central air conditioning could increase human exposure to air pollutants (Wing et al. 2000). Studies conducted in Germany and the United States reported that neighbors describe odors from swine CAFOs as annoying and offensive (Schiffman 1998; Tajik et al. 2008; Thu 2002, 2003; Thu and Durrneberger 1998; Radon et al. 2007). In a previous study of communities neighboring North Carolina CAFOs (Schinasi et al. 2011), we found that self-reported hog odor and H_2_S are associated with acute irritation of the eyes, nose, and throat, and also that particulate matter ≤ 10 µm in aerodynamic diameter (PM_10_) is associated with eye irritation. In addition to physical symptoms and negative mood (Bullers 2005; Horton et al. 2009; Schiffman et al. 1995), CAFO neighbors have reported that because of frequent and unpredictable episodes of malodor, they were unable to engage in valued traditions of rural life, including gardening, family gatherings, cookouts, visiting neighbors, and drying laundry (Tajik et al. 2008; Thu 2002, 2003; Thu and Durrneberger 1998).

Several studies have found relationships between malodor from swine CAFOs and chronic (Schiffman et al. 1995) or acute (Horton et al. 2009) stress in neighbors. Other studies have reported that environmental stressors are associated with increased blood pressure (BP) (Attarchi et al. 2012; Belojevic and Evans 2012; Djindjic et al. 2012) and that odorant compounds perceived as pleasant attenuated exercise-related increases in BP (Nagai et al. 2000). African Americans and low-income people experience an excess prevalence of chronic hypertension (Carson et al. 2011; Keenan and Rosendorf 2011; Liao et al. 2011), as well as hypertension-related morbidity (Liao et al. 2011) and mortality (Fiscella and Holt 2008). Identification of environmental factors that contribute to BP elevations could inform efforts to prevent upward shifts of BP in populations.

In this study we evaluated whether measures of swine CAFO air pollution were associated with acute changes in BP among neighbors during follow-up of approximately 2 weeks. We did not compare BPs of CAFO neighbors and other people; rather, we compared each participant’s BP during times of more and less exposure to swine CAFO air pollution. In this design each participant served as her or his own control. Characteristics that were essentially constant during the short follow-up (e.g., age, socioeconomic status, medical history, body mass, occupation, personality) could not cause bias in estimates of the exposure–outcome relationship. Chronic effects of exposure, however, could not be evaluated.

## Methods

*Setting and data collection.* The study was conducted in partnership with the Concerned Citizens of Tillery (CCT), a community-based organization in Halifax County that promotes the health, environmental, and political interests of predominantly African-American communities in eastern North Carolina (Wing et al. 1996). CCT has partnered with universities to provide medical care through the Tillery People’s Clinic and to conduct research on health and environmental justice (Tajik and Minkler 2006). For this study, the CCT staff organized community meetings in areas with a high density of swine CAFOs and provided information about our ongoing study to attendees, who were invited to contact CCT or University of North Carolina–Chapel Hill researchers if they were interested in participating in the study (Wing et al. 2008a). We sequentially enrolled between 4 and 10 volunteers in each of 16 rural communities from 2003 to 2005, and participants began data collection within 24–36 hr. Enrollment did not take place between mid-December and mid-February because of holidays and cold weather. Numbers of nearby swine CAFOs, participants, and other community-specific characteristics have been reported previously (Wing et al. 2008b).

To be eligible, participants had to be ≥ 18 years of age and nonsmokers, and live within 1.5 miles of at least one swine CAFO (Wing et al. 2008a), defined as a facility housing > 250 animals and using a liquid waste management system (Wing et al. 2000). At an initial training session, participants chose morning and evening times when they would sit outside each day for approximately 2 weeks (in three neighborhoods participants chose to continue up to 1 more week). They provided information about regular use of medications, and each participant’s odor sensitivity was tested using a standard set of butanol dilutions to evaluate the lowest concentration that could be distinguished from zero (e.g., Croy et al. 2009). Participants completed the John Henryism Active Coping (JHAC) scale, which measures the predisposition to respond behaviorally to psychosocial environmental stressors (James et al. 1987); higher values indicate a greater predisposition to cope actively. Participants were classified by reported use (yes/no) of antihypertensive medications (e.g., drugs classified as beta blockers, calcium channel blockers, angiotensin-converting-enzyme inhibitors, diuretics). They learned how to use a structured diary to record levels of swine odor, stress, and symptoms, and they practiced measuring their BP with an automated oscillometric device. Time spent outdoors and times of diary completion were tracked using a digital clock provided and set by researchers. Informed consent was obtained at the training session using a procedure approved by the University of North Carolina Institutional Review Board, which reviewed the study annually. We obtained a Certificate of Confidentiality from the National Institutes of Health (Wing et al. 2008a) because of prior attempts by the pork industry to obtain confidential records (Wing 2002).

Each morning and evening, participants sat outside for 10 min and completed the first of four pages of a data-collection diary. They then returned indoors to complete the remaining pages and measure their BP (Wing et al. 2008a). They rated the strength of swine odor during the 10-min period outdoors on a nine-level Likert-type scale [0 (none) to 8 (very strong)], and evaluated perceived stress (“How do you feel now … stressed or annoyed?) on a nine-level scale [0 (none) to 8 (extremely)]. Participants measured their BP twice in a seated position. They were instructed to wait 1 min between readings, raising their right arm above their head for the first 30 sec and then resting for the remaining time before taking their BP again. They printed the results and taped the printout with the systolic (SBP) and diastolic (DBP) values and current time into the diary. We treated the average of the two readings as dependent variables.

While participants collected data, we monitored air pollution at a central location in each neighborhood. The mean and median distance from air monitors to participant homes was 0.2 miles and 0.1 miles, respectively (Wing et al. 2008a). Swine CAFOs release many odorant chemicals including ammonia, H_2_S, and hundreds of volatile organic compounds (Schiffman et al. 2001). Odorant chemicals may occur as gases or particles. We quantified H_2_S, which is produced by the anaerobic decomposition of fecal waste, as a marker of this complex mixture that is related to hog odor intensity (Wing et al. 2008b; Schiffman et al. 2005). H_2_S is a specific marker of swine CAFO pollution in the study areas because other H_2_S-emitting industries such as waste water treatment plants, petrochemical plants, and paper mills, were not present. Average ambient H_2_S concentrations measured every 15 min with an MDA Scientific Single Point Monitor (Zellweger Analytics Inc., Lincolnshire, IL) were used to calculate hourly averages; 15-min values below the detection limit of 2 ppb were treated as zero. We considered average concentrations during the 1 hr before BP measurements as predictors of SBP and DBP.

We measured hourly levels of PM_10_ using a Series 1400a tapered element oscillating microbalance Ambient Particulate Monitor (Rupprecht and Patashnick Co. Inc., East Greenbush, NY). A Series 8500 FDMS Filter Dynamics Measurement System (Rupprecht and Patashnick Co. Inc.) was used to quantify semivolatile PM_10_. Semivolatile particles consist of compounds that are present in both vapor and condensed phases. Airborne PM is ubiquitous; although CAFOs are one source, particles are not a specific marker of CAFO pollutants. We reported previously that semivolatile PM_10_ showed little association with hog odor in the study neighborhoods and that PM_10_ was related to hog odor only when wind speeds were high (Wing et al. 2008b).

*Statistical analysis.* In this repeated-measures design, each participant served as her or his own control. The sample size is a function of the number of participants and the number of observations (records) per person. We used linear fixed-effects regression to model repeated measures for individuals (Allison 2005). This approach estimates the average within-person associations between exposure measures and BP by conditioning on person, and eliminates bias from any measured or unmeasured confounding factors that do not change during follow-up. Relationships between SBP and DBP and air pollution appeared linear across categories of exposure (data not shown), so they were modeled as continuous variables. BP varies diurnally, as do hog odor and H_2_S (Wing et al. 2008b); therefore, time of day (AM vs. PM) was included as a covariate in all models. In separate analyses, we also adjusted for self-reported stress, a potential mediator of associations between pollutants and BP. Sex and odor detection threshold (dichotomized at the median) were considered potential modifiers related to odor perception, whereas JHAC score (dichotomized at the median) and use of antihypertensive medication (yes/no) were considered potential modifiers of BP reactivity to environmental stressors. We also considered modification by age (dichotomized at the median) because it could influence either odor perception or BP reactivity.

Observations (records) with missing values for a variable were dropped from models including that variable. Model coefficients represent the average within-person change in BP for each unit increase in pollution. In nonrandomized studies, confidence limits and *p*-values do not quantify the confidence or probability that a point estimate would occur within a specified interval due to chance; therefore, we report standard errors of the regression coefficients as a measure of precision and *t*-values as indicators of the improvement in the fit of the model associated with the exposure variable. Degrees of freedom for *t*-tests, *n*-1, are large and can be considered equivalent for comparing *t* values.

## Results

Descriptive characteristics of the 101 participants are given in Table 1. Half of the participants were > 53 years of age, and two-thirds were women. Among the 97 participants whose odor detection threshold was determined, 55 had a butanol odor detection threshold of ≤ 40 ppm. Forty-two participants reported taking one or more BP medications. Among the 96 participants who completed the JHAC, 46 had a score > 52. Most participants (85) identified themselves as black.

**Table 1:** Characteristics of participants [*n* (%) of nonmissing observations], Community Health Effects of Industrial Hog Operations study.

Variable	Participants (N = 101)	Records (N = 2,949)
Age (years)				
≤ 53.7	51 (50.5)	1,410 (47.9)
> 53.7	50 (49.5)	1,539 (52.2)
Gender				
Women	66 (65.3)	1,945 (66.0)
Men	35 (34.7)	1,004 (34.0)
Odor threshold				
Missinga	4 (4.0)	91 (3.1)
Butanol ≤ 40ppm	55 (56.7)	1,559 (54.5)
Butanol > 40ppm	42 (43.3)	1,299 (45.5)
BP medication used				
No	59 (58.4)	1,680 (57.0)
Yes	42 (41.6)	1,269 (43.0)
JHAC scoreb				
Missinga	5 (5.0)	117 (4.0)
≤ 52	50 (52.1)	1,480 (52.3)
> 52	46 (47.9)	1,352 (47.7)
aPercent of all observations. bHigher JHAC score indicates higher active coping with psychosocial stressors.				

Table 2 presents distributions of reported hog odor intensity during the 10 min outdoors, average pollutant concentrations in the hour before BP measurement, SBP, and DBP. Odor ratings were missing in 6% of the records, and no odor was reported in 48% of the records. Very strong odor (a rating of 6, 7, or 8) was reported 6% of the time. Hourly H_2_S measurements were missing in approximately 9% of the records, and most (88%) were below the limit of detection (2 ppb). PM measures were missing in 32.2% of the records, primarily because of equipment malfunction during periods of high temperature and humidity (Wing et al. 2008b). For 12.4% of records, semivolatile particle concentrations were < 0; this occurs when concentrations are low because microbalance estimates are derived by subtraction of sequential mass values that are measured with error (Wing et al. 2008b). BP was missing in 1.4% of the records. SBP readings were < 120 mmHg in approximately 30% of the records and > 140 mmHg in approximately 25% of the records. DBP was < 80 mmHg in 61% of the records and ≥ 90 mmHg in 11% of the records. No participants were missing data for all their records.

**Table 2:** Distributions of odor, H_2_S, and BP from the total of nonmissing records (*N* = 2,949), Community Health Effects of Industrial Hog Operations study.

Variable (scale)	n (%)	
Odor (0–8)
Missinga	177	(6.0)
None	1,419	(48.1)
1–2	779	(26.4)
3–5	407	(13.8)
6–8	167	(5.7)
Stress (0–8)
Missinga	58	(2.0)
None	2,331	(80.6)
1–2	436	(15.1)
3–5	91	(3.2)
6–8	33	(1.2)
H2S (ppb)
Missinga	255	(8.6)
0	2,412	89.5
0–2	170	(6.3)
2–4.99	77	(2.9)
5–47.5	35	(1.3)
PM10 (µg/m3)
Missinga	948	(32.1)
< 10	415	(20.7)
10–19.9	783	(39.1)
20–29.9	528	(26.4)
30–502.0	275	(13.7)
Semivolatile PM10 (µg/m3)
Missinga	948	(32.2)
< 0	366	(18.3)
0–2.99	638	(31.9)
3–7.99	767	(38.3)
> 8	230	(11.5)
SBP (mmHg)
Missinga	41	(1.4)
< 120	897	(30.8)
120–139	1,257	(43.2)
140–159	510	(17.5)
> 160	244	(8.4)
DBP (mmHg)
Missinga	41	(1.4)
< 80	1,804	(62.0)
80–89	781	(26.9)
90–99	221	(7.6)
> 100	102	(3.5)
aPercent of all records.		

Associations between air pollutants and BP adjusted for time of day (AM or PM) are presented in Table 3. Each unit increase in reported hog odor on the 0–8 intensity scale was associated with average estimated increases [β (SE)] of 0.10 (0.12) and 0.23 (0.08) mmHg for SBP and DBP, respectively. A 1-ppb increase in H_2_S was associated with increases of 0.29 (0.12) mmHg for SBP and 0.12 (0.08) mmHg for DBP. PM_10_ was not associated with BP. Semivolatile PM_10_ was not associated with SBP but had a small negative association with DBP [–0.06 (0.03)].

**Table 3:** Linear fixed effects beta coefficients (SEs) and t-values for associations of one-unit increases in pollutants with SBP and DBP, adjusted for time-of-day (AM or PM), Community Health Effects of Industrial Hog Operations study.

Pollutant	SBP			DBP		
	β (SE)	t-Value	β (SE)	t-Value
Odor (0–8)	0.10 (0.12)	0.86	0.23 (0.08)	3.02
H2S (ppb)	0.29 (0.12)	2.45	0.12 (0.08)	1.52
PM10 (µg/m3)	–0.01 (0.01)	–0.78	–0.00 (0.01)	–0.41
Semivolatile PM10 (µg/m3)	–0.02 (0.05)	–0.45	–0.06 (0.03)	–1.66

Table 4 provides beta coefficients for hog odor and H_2_S according to potential modifying variables. Coefficients for PM_10_ and semivolatile PM_10_ are not shown because their main effect estimates were small, they are not specific markers of swine CAFO air pollution, and data are missing for almost one-third of the records. Hog odor coefficients for SBP were all positive, but none had *t*-values > 1.17. Coefficients for DBP were positive and all had *t*-values near or above 2 except for participants ≤ 53.7 years of age, for whom the β (SE) is 0.08 (0.12). Coefficients for both SBP and DBP were larger for older participants than younger participants [0.14 (0.15) and 0.33 (0.10) vs. 0.04 (0.18) and 0.08 (0.12), respectively] and for men than women [0.20 (0.23) and 0.36 (0.15) vs. 0.07 (0.13) and 0.19 (0.09), respectively]. Associations between hog odor and SBP were larger for participants with JHAC scores ≤ 52 compared with those for persons with JHAC scores > 52 [0.18 (0.17) compared with 0.01 (0.16)] and for participants who reported no use of antihypertensive drugs compared with those with regular use [0.19 (0.16) compared with 0.01 (0.17)]. For H_2_S, coefficients for both SBP and DBP were larger for men than women [0.56 (0.30) and 0.48 (0.19) compared with 0.24 (0.13) and 0.05 (0.08), respectively]; participants with butanol odor sensitivity thresholds > 40 ppm than for those with thresholds ≤ 40 ppm [0.33 (0.14) and 0.13 (0.09) compared with 0.17 (0.22) and 0.07 (0.14), respectively]; and participants with JHAC scores of ≤ 52 than those with scores > 52 [0.36 (0.14) and 0.17 (0.09) compared with 0.02 (0.24) and –0.07 (0.15), respectively]. The SBP coefficient was larger for participants who did not report taking BP medications compared with those who did [0.38 (0.14) compared with 0.07 (0.22)].

**Table 4:** Linear fixed effects beta coefficients (SEs) and t-values for potential modifiers of associations of BP with one-unit increases in hog odor and H_2_S, adjusted for time-of-day (AM or PM), Community Health Effects of Industrial Hog Operations study.

Modifier	SBP			DBP		
	β (SE)	t-Value	β (SE)	t-Value
Hog odor (0–8)
Age ≤ 53.7 years	0.04 (0.18)	0.23	0.08 (0.12)	0.68
Age > 53.7 years	0.14 (0.15)	0.93	0.33 (0.10)	3.34
Women	0.07 (0.13)	0.50	0.19 (0.09)	2.11
Men	0.20 (0.23)	0.85	0.36 (0.15)	2.37
Butanol threshold ≤ 40 ppm	0.10 (0.15)	0.67	0.21 (0.10)	2.17
Butanol threshold > 40 ppm	0.10 (0.19)	0.54	0.24 (0.12)	2.03
JHAC score ≤ 52	0.18 (0.17)	1.07	0.22 (0.11)	2.05
JHAC score > 52	0.01 (0.16)	0.06	0.20 (0.11)	1.92
No BP meds	0.19 (0.16)	1.17	0.25 (0.11)	2.31
Any BP meds	0.01 (0.17)	0.04	0.21 (0.11)	1.96
H2S (ppb)
Age ≤ 53.7 years	0.30 (0.15)	1.97	0.13 (0.10)	1.32
Age > 53.7 years	0.28 (0.19)	1.45	0.10 (0.12)	0.78
Women	0.24 (0.13)	1.85	0.05 (0.08)	0.58
Men	0.56 (0.30)	1.90	0.48 (0.19)	2.51
Butanol threshold ≤ 40 ppm	0.17 (0.22)	0.78	0.07 (0.14)	0.48
Butanol threshold > 40 ppm	0.33 (0.14)	2.40	0.13 (0.09)	1.49
JHAC score ≤ 52	0.36 (0.14)	2.67	0.17 (0.09)	1.90
JHAC score > 52	0.02 (0.24)	0.08	–0.07 (0.15)	–0.45
No BP medication	0.38 (0.14)	2.70	0.10 (0.09)	1.12
Any BP medication	0.07 (0.22)	0.34	0.15 (0.14)	1.07

SBP and DBP were strongly associated with reported stress, increasing on average 0.82 (0.21; *t* = 3.98) and 0.57 (0.13 mmHg; *t* = 4.28), respectively, for every unit increase on the 0–8 scale. We included stress in models reported above (in addition to time of day) to evaluate whether associations of BP with hog odor and H_2_S change after adjustment for this potential mediator. With adjustment for reported stress, coefficients for the association between hog odor and DBP declined from 0.23 (0.08) to 0.15 (0.08), whereas the coefficient for SBP decreased from 0.10 (0.12) to –0.04 (0.12). With adjustment for reported stress, there was little change in the coefficient for the association between H_2_S and DBP [0.15 (0.08) vs. 0.12 (0.08) before adjustment] or SBP [0.26 (0.12) vs. 0.29 (0.12) before adjustment].

## Discussion

In this community-based participatory repeated-measures study we found that, on average, BP of participants living near swine CAFOs increased in association with increases in markers of transient plumes of odorant air pollution. Because each participant served as her or his own control, factors that did not change during the 2-week study—including body mass, race, socioeconomic position, medical and dietary history, and prior BP—​could not confound these associations. Estimated DBP was almost 2 mmHg higher during periods of very strong odor (a rating of 8) compared to none, and estimated SBP was almost 3 mmHg higher when H_2_S concentrations were 10 ppb compared with times when H_2_S was zero (below the limit of detection). This magnitude of effect could have public health importance because of the frequency and duration of odor episodes near CAFOs. The 101 people who participated in this study for approximately 2 weeks reported 1,655 episodes of outdoor hog odor, 38% of which lasted > 1 hr, and 17% of which had a mean odor ≥ 5 (on the scale of 0–8); participants also reported 500 episodes of indoor odor (Wing et al. 2008b). If the associations were causal and if malodors from other sources such as sewage, landfills, and chemical refineries produce similar effects, then control of environmental malodor might help prevent repeated acute elevations of BP that could contribute to development of chronic hypertension.

With approximately 29 measures per person, the sample size for this study was primarily suited to examining within-person covariation in exposures and outcomes. Although estimates within subgroups defined by non–time-varying factors are imprecise, some interactions are of interest. Associations between H_2_S and SPB were similar for both older and younger participants, whereas the odor–DBP association was observed primarily among older participants. Beta coefficients for both odor and H_2_S were larger for men than women. The magnitude of the association between BP and hog odor was not related to the butanol odor sensitivity threshold. Because the effectiveness of peoples’ active coping is reduced by lack of resources, persons with high JHAC scores and low socioeconomic position are expected to be more physiologically reactive to psychosocial stressors than people with high JHAC scores and high socioeconomic position, or people with low JHAC scores (James et al. 1987). Contrary to our expectation, even though all participants in this study lived in low-income areas, associations between hog air pollution markers and BP were not stronger among participants with high JHAC scores. Associations for SBP were generally weaker among participants who were taking BP medications, which may reduce responses to environmental stimuli.

Although the repeated-measures design and fixed-effects analysis precludes confounding from time-independent factors that differ between people, time-related factors associated with both air pollution and BP could have either attenuated or exaggerated associations. Time of day (AM vs. PM) was included in all models; therefore, potential time-related factors would need to be associated with pollution and BP within times of day in order to act as confounders. Time-related confounding could occur if a cause of acute BP change that is not a consequence of CAFO air pollution covaried with the CAFO air pollutants in participants’ neighborhoods.

Measurement errors could also impact estimates of association between odorant pollutants and BP. In a clinical or experimental setting, BP is typically measured by a trained technician in a standardized manner. In contrast, in the present study, each participant measured her or his own BP twice each day at home, which could reduce the precision of the effect estimates. Use of a portable printer with a time stamp to record BP values in the diaries prevented transcription errors that could have introduced systematic errors related to odor intensity. The temporal sequence of sitting outside prior to BP measurement was reversed in < 2% of records (Schinasi et al. 2009).

Although participants recognized hog odor and could rate it on the 0–8 scale from “none” to “very strong,” we did not evaluate the reproducibility of their ratings, which could be affected by physical and social context. For example, participants might rate an odor as more intense on a day that they expected company if they were ashamed of their expected guests’ reactions to the presence of fecal odor at their home. More precise measures of odor can be made in units of dilution to threshold using an olfactometer (Lambert et al. 2000); however, it was not feasible to use such a device in this participatory study. We evaluated participants’ odor sensitivity threshold using a butanol standard and expected that associations between hog odor and BP might be attenuated among participants with poorer odor sensitivity; however, associations with hog odor differed little by odor sensitivity. In an experiment including 44 volunteers, van Thriel et al. (2008) reported that butanol odor threshold was not related to ratings of environmental odorants.

H_2_S was the chemical marker of odorant swine CAFO air pollution that we could quantify over short time period; these measures cannot be affected by response bias. Because there are no other major industrial sources of H_2_S in the study communities, it is a specific marker of swine CAFO emissions; however, this marker is not sensitive, in part, because of the detection threshold of the instrument (~ 2 ppb/15 min). Hog odor, which has a distinctive character due to a complex mixture of volatile organic compounds (Schiffman et al. 2001; Karageorgos et al. 2010), was often reported when H_2_S levels were below the detection limit. Another source of measurement error comes from the placement of the H_2_S monitor at a central location in rural neighborhoods, which was as far as approximately 1 mile from some participants’ residences (median, 0.1 mile). Narrow plumes of odorant compounds from swine CAFOs could be present at participants’ homes but not at the monitor, or vice versa. We expect this type of exposure misclassification would attenuate any real associations between H_2_S and BP.

Relationships between odorant air pollutants and BP could be produced by psychophysiological or pharmacological mechanisms (Shusterman 1992). Our findings that odor and H_2_S, but not PM, were associated with BP increases are consistent with a psychophysiological mechanism. The lack of an association with PM could also be related to the lower levels or different composition of PM in rural communities compared with urban areas typically studied. Furthermore, many observations were missing for PM. We evaluated BP in this study because environmental exposure to swine odor in this population has been associated with self-reported stress (Horton et al. 2009), and acute stress is associated with transient BP elevation (Sparrenberger et al. 2009). Odorant pollution could also produce other changes in a person’s environment that cause acute changes in BP, for example, irritability of a household member.

The pharmacological actions of swine CAFO air emissions on BP are unknown and difficult to predict because emissions include many chemical compounds and fine particles (Schiffman et al. 2001). Although we measured H_2_S as an indicator of the odorant component of this mixture, growing evidence suggests that H_2_S, an endogenous gasotransmitter, acts as a vasodilator (Wagner 2009). To the extent that exogenous H_2_S plays a similar role, its presence in odorant plumes could therefore attenuate associations between swine odor and BP.

The setting for our study, the coastal plain of eastern North Carolina, has one of the highest densities of swine production in the world (Pew Commission on Industrial Food Animal Production 2008). Historically, it is part of both the Black Belt (home to a majority of rural African Americans) and the stroke belt (an area of high mortality from cerebrovascular and cardiovascular diseases) (Casper et al. 1995). Swine CAFOs in the state are highly disproportionately located in low-income communities of color (Wing et al. 2000). If swine CAFO air pollution contributes to high BP in this region, the associated cardiovascular morbidity and mortality would be among the consequences of environmental injustice.

Malodors are produced by other types of CAFOs, waste disposal sites, refineries, chemical plants, waste water treatment plants, and land application of sewage sludge. These facilities and activities expose communities that lack political power to environmental malodors while benefiting consumers and producers in nonimpacted areas. Therefore, the generalizability of findings reported here is relevant to public health protection. Communities with low levels of political influence are less able to prevent siting of such facilities than are communities with political power, and they are less able to demand the best technologies for reducing resulting pollutants. Repeated acute physical environmental stressors, such as malodor and noise, may be aspects of the built environment that contribute to racial and economic disparities in high BP and its sequelae.
